# Asymmetric gap balancing improves knee kinematic following primary total knee arthroplasty

**DOI:** 10.1186/s42836-024-00243-5

**Published:** 2024-04-25

**Authors:** Pieralberto Valpiana, Andrea Giordano Salvi, Stefano Ghirardelli, Matteo Bernardi, Giuseppe Petralia, Giuseppe Aloisi, Christian Schaller, Pier Francesco Indelli

**Affiliations:** 1 Südtiroler Sanitätsbetrieb, 39042 Brixen, Italy; 2 Institute for Biomedicine, EURAC Institute, 39100 Bozen, Italy; 3 Personalized Arthroplasty Society (PAS), One Glenlake Parkway NE, Suite 1200, Atlanta, GA 30328 USA; 4 Institute of Biomechanics, Paracelsus Medical University (PMU), 5020 Salzburg, Australia; 5 Department of Clinical Medicine, Public Health, Life and Nature, University of L’Aquila, P.Le S. Tommasi 1, 67100 L’Aquila, Italy; 6 The Breyer Center for Overseas Studies, Stanford University in Florence, 50125 Florence, Italy; 7 “Paolo Aglietti” Gait Lab, CESAT, Azienda Sanitaria Toscana Centro, 50054 Fucecchio, Italy

**Keywords:** TKA, Medial pivot, Gait analysis, Robotics, Kinematic alignment

## Abstract

**Purpose:**

The purpose of this study was to demonstrate closer-to-normal knee kinematics following primary total knee arthroplasty (TKA) performed establishing asymmetric gap balancing intraoperatively.

**Material and method:**

Two age-, sex-, BMI-matched groups of patients underwent medially stabilized TKA because of isolated knee disease. Group A (12 patients) underwent “unrestricted” kinematic alignment (uKA) according to Howell while group B (15 patients) received robot-assisted “simplified” KA (sKA) with an alignment goal (Hip-Knee-Ankle axis-HKA) ± 5° respect to the mechanical axis. Intraoperatively, in group B, the flexion gap at 90° was first set at an average of 1.5 mm (0–5 mm; SD 4.4 mm) tighter in the medial compartment with respect to the lateral; in the same way, the extension gap was then set at an average of 2.0 mm (0–4.5 mm; SD 3.1 mm) tighter in the medial compartment with respect to the lateral. All patients, including a non-arthritic cohort (group C: 5 controls) underwent gait analysis using an instrumented treadmill (WalkerView–WV) equipped with an instrumented belt armed with a 3D video camera. The WV software evaluated multiple spatiotemporal and kinematic parameters, including: (1) contact time (s); (2) knee ROM during gait cycle; (3) step length percentage with respect to total gait (%) and pure step length (cm). Statistical analyses included *t*-Test and ANOVA and were conducted by using SPSS.

**Results:**

At the final FU, significant differences were noted during gait between the two TKA groups (uKA-sKA) and the controls. Both TKA groups showed superior mean contact time on the surgical knee (uKA 1 s; sKA 0.97 s) as compared to the controls (0.72 s) (*P* = 0.002) while no differences were found between them (*P* = 0.11). TKA groups showed a lower, maximum ROM in the surgical knee (mean uKA 36º; mean sKA 49º) relative to the controls (mean 57º) (*P* < 0.05) but a statistical difference was found between them (*P* = 0.003). Both TKA groups showed a higher step length percentage with respect to the total gait and a shorter step length on the surgical side (uKA: mean 8.28% and mean step length 35.5 cm; sKA: mean 8.38% and mean step length 34.6 cm) in comparison to the controls (mean 3.38%; mean step length 71.4 cm) (*P* < 0.05) while no statistical differences were found between them.

**Conclusion:**

To our knowledge, this was the first study to exhibit the kinematic advantages of a slightly asymmetric gap balancing during KA TKA. Combining a medially-stabilized implant design and a surgical technique aiming to obtain a tighter medial compartment represents a promising approach to improve outcomes after TKA.

**Graphical Abstract:**

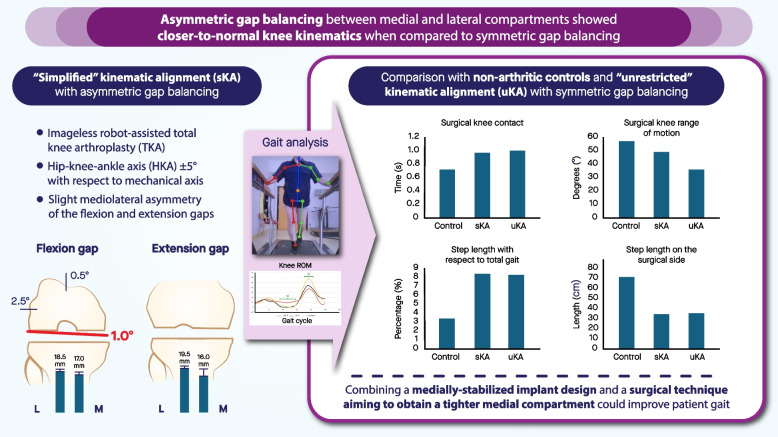

## Introduction

Historically, normal knee kinematics has not been reproduced following total knee arthroplasty (TKA) even when cruciate sparing designs and a mechanical alignment (MA) surgical technique were intraoperatively selected [1].

Both surgical techniques historically used to balance a TKA (“measured resection” and “gap balancing”) aimed for a perfect, symmetric balancing between the lateral and medial knee compartments [2]. Unfortunately, it has been shown that the recreation of symmetric and rectangular spaces in flexion and extension [3] does not match the kinematic profile of the native knee [4].

We and some other authors challenged the dogma that achieving symmetric gaps during the balancing phase of the TKA procedure should still represent the “gold standard” [5, 6]. Thanks to the introduction of enhanced digital technologies, such as robotics, smart sensors, and augmented reality tools, the empirical and surgeon-dependent definition of a well-balanced knee can be finally replaced by the accuracy in gap measurements yielded by computer-assisted technologies. However, an exact quantification of the desirable amount of inter-compartmental asymmetry during the TKA procedure is still lacking. This is also because gait analysis studies comparing symmetrically-balanced and asymmetrically-balanced knees are lacking. The fear of mid-flexion instability also pushed surgeons to stay in the “safe zone” of symmetric gap balancing since surgical technique-related risk factors have been believed to be responsible for this complication [7].

We recently published a robot-assisted, simplified surgical technique [8], which was based on the recreation of clearly defined, slightly asymmetric flexion and extension gaps. This surgical technique was combined with the use of a medially-stabilized TKA design. This gait analysis study hypothesized that the robotic-guided, intraoperative reproduction of a slight mediolateral gap asymmetry during TKA improved the postoperative knee kinematics compared to the traditional inter-compartmental gap symmetry.

## Materials and methods

Two retrospectively matched (age, sex, BMI) groups of patients underwent medially-stabilized TKA because of isolated knee disease. Patient demographics are shown in Table 1. Inclusion criteria in both TKA groups were: (1) age > 18 years, (2) successful TKA with Knee Society Clinical Score > 80, and (3) preoperative, unilateral bicompartmental knee OA. Exclusion criteria included the presence of chronic inflammatory diseases or other disorders affecting the execution of the gait analysis. Group A (12 patients) underwent “unrestricted” kinematic alignment (uKA) with symmetric gap balancing according to Howell et al. [9]; the same medial pivot implant (GMK Sphere, Medacta, Castel San Pietro, Switzerland) was used in all cases. Group B (15 patients) underwent robot-assisted “simplified” kinematic alignment (sKA) with an alignment goal (Hip-Knee-Ankle axis-HKA) ± 5° with respect to mechanical axis and a slight mediolateral asymmetry of the flexion and extension gaps [8]. The same medially-congruent implant (Persona MC, Zimmer Biomet, Warsaw, IN, USA) was employed in all cases. In group B, the flexion gap at 90° was set first at an average of 1.5 mm (0–4.5 mm; SD 3.1 mm) tighter in the medial compartment with respect to the lateral. In the same way, the extension gap was then set at an average of 2 mm (0–5 mm; SD 4.4 mm) tighter in the medial compartment with respect to the lateral. All surgeries were performed under spinal anesthesia with the use of a tourniquet.

**Table 1:** Demographics of the study groups

	**Control Group**	**uKA**	**sKA**	***P*** **-Value**
Sample size (*n*)	5	12	15	N/A
Gender	60% M; 40% F	66% M; 44% F	53% M; 47% F	0.89
Age (years)	26.6	78.1	74.2	2.11
Weight (kg)	73.2	82.7	81.1	0.39
BMI (kg/m^2^)	23	27.5	26.3	1.02

*uKA* unrestricted kinematic alignment [9], *KA* kinematic alignment, *sKA* simplified kinematic alignment [8], *BMI* Body Mass Index, *F* female, *M* male, *N/A* not applicable. Data are reported as average

All TKA patients were radiologically evaluated before gait analysis. The final LDFA, MPTA, and final HKA were measured on weight-bearing, full-leg films to correlate the final HKA axis to the gait analysis data.

All patients, including a non-arthritic cohort (Group C: 5 healthy controls) underwent gait analysis evaluation. The gait analysis was carried out utilizing a modern instrumented treadmill (WalkerView™-WV-by TecnoBody, Dalmine, Italy) equipped with an instrumented belt enriched with 8 load cells, a 48"-wide LCD screen providing continuous virtual reality/biofeedback, a 3D video camera (Kinect v2, Microsoft, USA) and a control, 15" touchscreen interfaced to PC. The WV-integrated software utilized for the gait analysis (TecnoBody MS, Dalmine, Italy) evaluated, in real-time fashion, multiple spatiotemporal parameters (cadence, stance/swing times, step time, and step length) and kinematic variables (spine, hips and knees ROM).

### Gait analysis setup and data processing

All TKA patients underwent gait analysis at a minimum follow-up (FU) of 9 months (270 days) from the surgical procedure. On average, the gait analysis was performed 289 days after the index procedure (range, 274–302 days). No patients underwent preoperative gait analysis. Before gait trials, healthy controls and patients were asked to familiarize themselves with the WV treadmill platform by undertaking a 20-min walk at their comfortable speed (maximum 20 km/h).

**Fig. 1:**
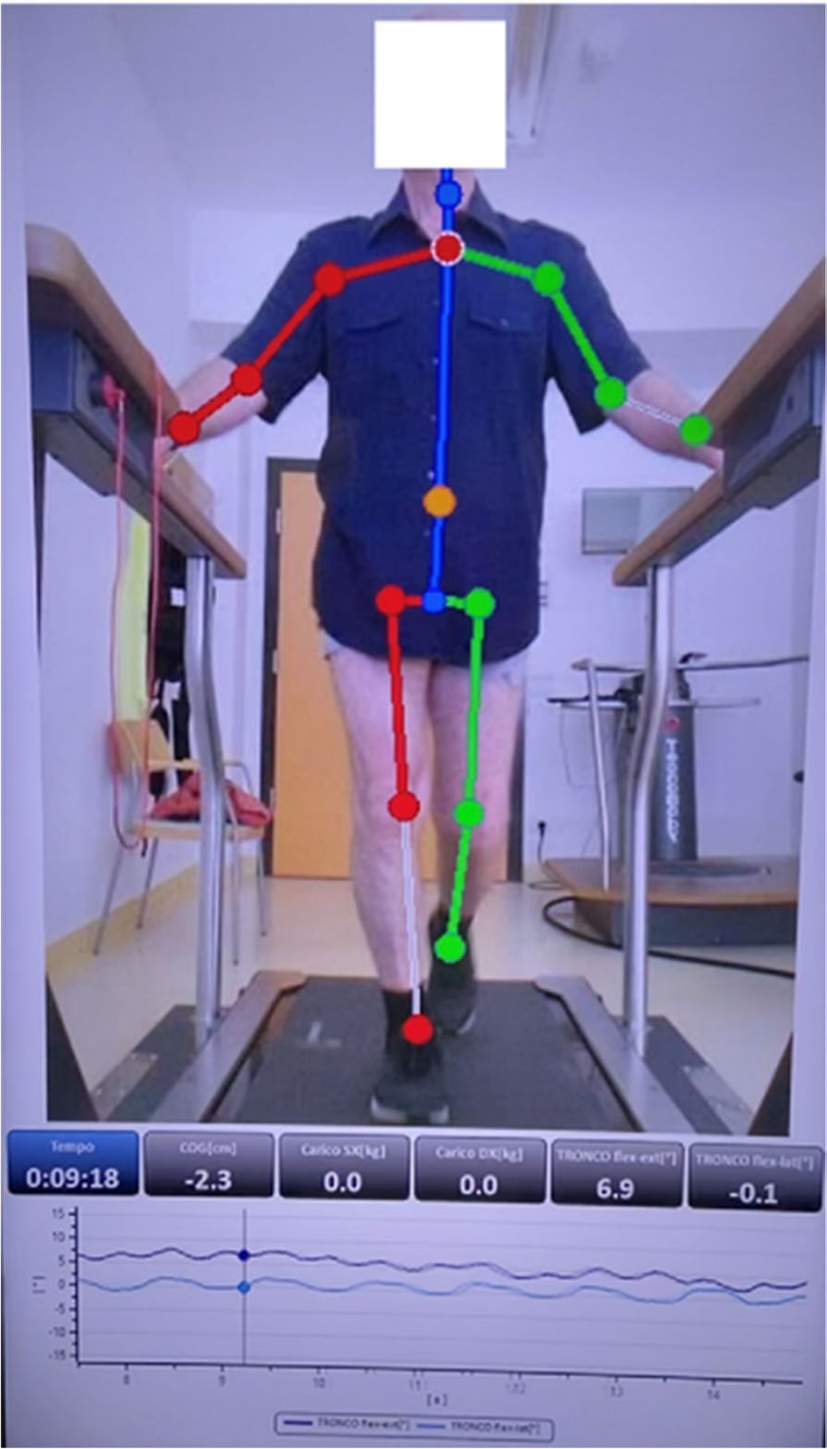
A 69-year-old patient undergoing gait analysis using an instrumented treadmill (WalkerView, TecnoBody MS, Dalmine, Italy)

After a 15-min trial, all participants underwent a 3-min gait test at their comfortable speed (Fig. 1). In particular, the belt speed was increased to the comfortable one gradually (about 30 s), then data of 2 min were captured, and lastly, the belt speed was gradually reduced to a stop (about 30 s). The recorded gait spatiotemporal and kinematic parameters were as follows: spine/hips/knees ROM; left/right load symmetry/asymmetry; cadence (cycle/s); left and right step length; time of ground contact; center of gravity variation during gait (cm and %).

Particular attention was paid to the following spatiotemporal and kinematic parameters: (1) Contact time (s); (2) Knee peak ROM during gait cycle; (3) Step length percentage with respect to total gait (%) and pure step length (cm). The same physical therapist (MB), with experience in gait analysis, oversaw the correct execution of the test and acquired all data.

### Statistical analysis

Spatiotemporal and kinematic parameters outcomes were compared using SPSS statistical package, version 25 (SPSS Inc, Chicago, IL, USA). Data from the three groups (control group, uKA group and simplified-KA group) were statistically compared for each variable of interest using student *t*-Test and one-way analysis of variance (ANOVA). The level of significance was set at *P* < 0.05. The study was approved by the internal review board (IRB: SABES 71/2023) and was performed according to Helsinki’s declaration.

## Results

A total of 32 gait trials were acquired: 5 in the control group, 12 in the uKA group, and 15 in the simplified KA group. In the control group, spatiotemporal and kinematic parameters were reported as an average between the left and right lower extremities, while, in the two TKA groups, data were reported and compared between the contralateral, healthy side, and surgical side.

### Spatiotemporal parameters

The main spatiotemporal findings are presented in Table 2. No statistical differences were found between the healthy controls and the two TKA groups other than in cadence and healthy knee step length (%). No statistical differences were found between the two TKA groups.

**Table 2:** Spatiotemporal parameters

Spatiotemporal Parameters	Control group	uKA group	Simplified KA group	*P*-Value
Cadence (cycle/s)	0.89 ± 0.5	0.72 ± 0.16	0.76 ± 0.10	uKA/sKA *P* = 0.36 Control/uKA/sKA *P* < 0.0003
Healthy side Step length (cm)	71.4 ± 4.4	33.8 ± 9.0	35.1 ± 13.7	uKA/sKA *P* = 0.59 Control/uKA/sKA *P* = 1.20
Surgery side Step length (cm)	NA	35.5 ± 9.1	36.3 ± 12.6	uKA/sKA *P* = 0.83
Healthy side Step length (%)	3.38 ± 0.9	7.4 ± 3.03	7.3 ± 3.8	uKA/sKA *P* = 0.71 Control/uKA/sKA *P* < 0.0004
Surgery side Step length (%)	NA	8.2 ± 3.4	7.8 ± 3.56	uKA/sKA *P* = 0.56
Healthy side Contact time (s)	0.72 ± 0.5	1.04 ± 0.3	0.92 ± 0.2	uKA/sKA *P* = 0.18 Control/uKA/sKA *P* = 0.052
Surgery side Contact time (s)	NA	1.03 ± 0.2	0.97 ± 0.2	uKA/sKA *P* = 0.48
Center of gravity Vertical Oscillation (cm)	3.08 ± 0.5	1.03 ± 0.2	1.26 ± 0.5	Control/uKA/sKA *P* = 2.6

Spatiotemporal parameters obtained during instrumented treadmill (WalkerView™–WV–by TecnoBody, Dalmine, Italy) testing. All the values are expressed as mean ± standard deviation, considering the values recorded from all individuals. Data from three groups (control group, uKA group and simplified-KA) were statistically compared for each variable of interest using one-way analysis of variance (ANOVA). Data from two groups (uKA group and simplified-KA group) were statistically compared for each variable of interest using student t-Test

*NA* not applicable, *uKA* unrestricted kinematic alignment [9], *KA* kinematic alignment, *sKA* simplified kinematic alignment [8]

### Kinematic parameters

**Fig. 2:**
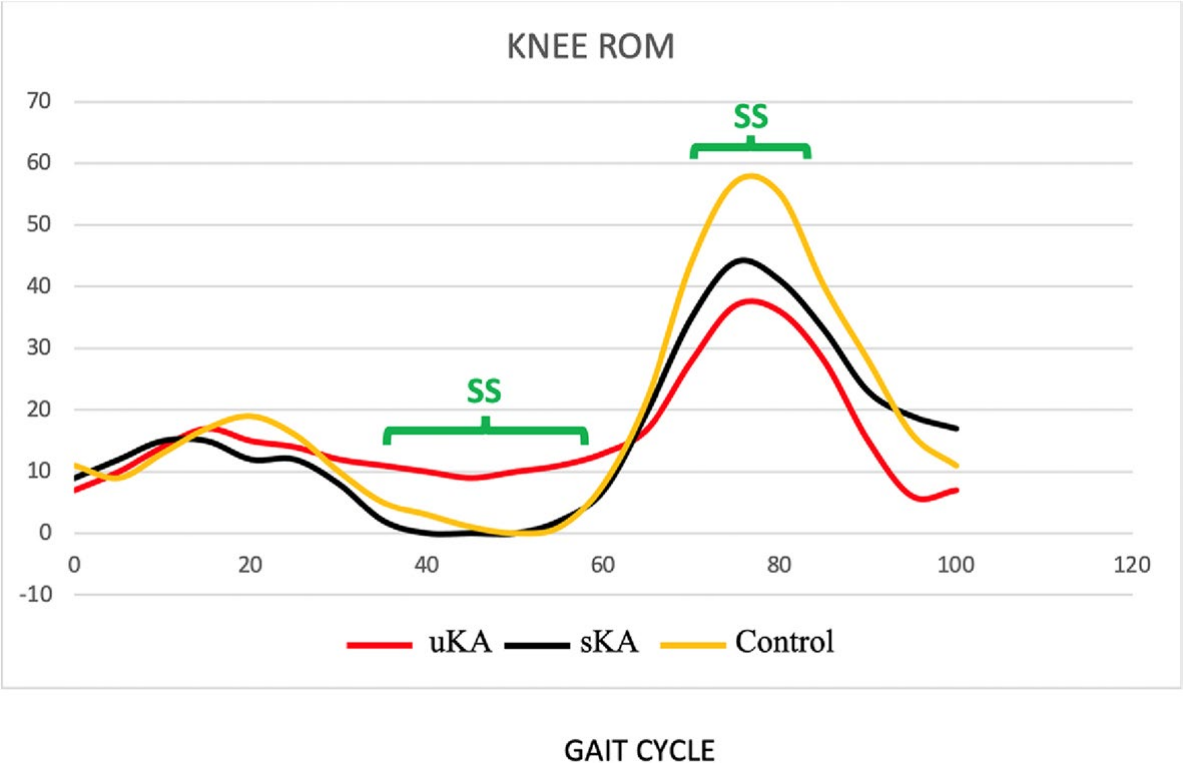
Knee range of motion (ROM) during the gait cycle: comparison between controls, uKA and simplified KA (sKA). sKA: simplified kinematic alignment [8]; uKA: unrestricted kinematic alignment. ROM was expressed as average values, and the gait curves have been extracted utilizing the software PlotDigitizer (Porbital, USA). SS: statistically significant differences were calculated using one-way analysis of variance (ANOVA)

The main kinematic findings are given in Table 3. Statistical differences were found between the healthy controls and the two TKA groups in several kinematic parameters, including anteroposterior (AP) spine flexion, and knee ROM (surgery side and healthy side). Statistical differences were found between the two TKA groups in AP spine flexion and knee peak ROM (surgery side and healthy side) (Fig. 2). A not statistically significant trend was also demonstrated between the two TKA groups in the peak hip ROM.

**Table 3:** Kinematic parameters

Kinematic Parameters	Control group	uKA group	Simplified KA group	*P*-Value
Spine AP Flexion (°)	4.2 ± 1.2	11.1 ± 3.7	8.5 ± 3.4	uKA/sKA *P* = 0.04 Control/uKA/sKA *P* = 0.0007
Spine ML Flexion (°)	0.71 ± 0.7	1.25 ± 1.0	0.86 ± 0.95	Control/uKA/sKA *P* = 0.31
Healthy Hip ROM (°)	50.3 ± 5.1	28.1 ± 5.5	32.2 ± 8.6	Control/uKA/sKA *P* = 3.41
Surgery side Hip ROM (°)	NA	31.3 ± 5	34.4 ± 7.9	Control/uKA/sKA *P* = 0.30
Healthy Knee ROM (°)	57.1 ± 3.1	34.1 ± 4	39.7 ± 11.5	uKA/sKA *P* = 0.10
Surgery side Knee ROM (°)	NA	33.6 ± 4.8	42.74 ± 8.5	uKA/sKA *P* = 0.0019

Kinematic parameters obtained during instrumented treadmill (WalkerView™–WV–by TecnoBody, Dalmine, Italy) testing. All the values are reported as mean ± standard deviation, considering the values recorded from all individuals. Data from three groups (control group, uKA group and simplified-KA) were statistically compared for each variable of interest using one-way analysis of variance (ANOVA). Data from two groups (uKA group and simplified-KA group) were statistically compared for each variable of interest using student T-test

* NA *not applicable, *uKA* unrestricted kinematic alignment [9], *KA* kinematic alignment, *sKA* simplified kinematic alignment [8], *AP* antero-posterior, *ML* medio-lateral, *ROM* range of motion (peak)

### Radiological evaluation

No statistical differences were found between the uKA and simplified KA groups in the final alignment (Table 4).

**Table 4:** Final lower extremities coronal alignment

	**uKA**	**Simplified KA (sKA)**
HKA Pre	175.8 (Varus) SD: ± 7.4	173.1 (Varus) SD: ± 3.9
HKA Post	178.5 (Varus) SD: ± 2.9	178.3 (Varus) SD: ± 1.7

Preoperative (Pre) and final (Post) Hip-Knee-Ankle (HKA) coronal alignments

*SD* Standard deviation, *uKA* unrestricted kinematic alignment [9], *sKA* simplified kinematic alignment [8]

## Discussion

To the authors’ knowledge, this was the first study to show the “in vivo” kinematic advantages of a slightly asymmetric intercompartmental gap balancing during primary TKA. When compared with simplified KA with slightly asymmetric gaps, patients following uKA with traditional symmetric gap balancing showed reduced knee flexion during the early stance and initial knee extension (i.e., midstance) phases of gait, reduced knee flexion during mid-swing and also higher hip extensor contribution to the total support moment during the early stance phase of gait. These findings confirmed previous reports [10], showing that individuals with less natural knee kinematics and joint instability demonstrated significantly reduced flexion and axial rotation knee motion excursions during the loading response phase of gait. Recently, Meneghini et al. [6] reported superior PROMs for patients with more lateral laxity, manually evaluated using a calibrated tensor device, at 90° of flexion compared with patients with more medial laxity. The authors [6] used a lateral conforming or a posterior-stabilized (PS) inserts, different from the current study where only medially-stabilized inserts were used. In the current study, the authors used the imageless robotic system in a navigation mode to accurately define the mediolateral asymmetry in the flexion and extension gaps [8]. It has been recently shown that enabling technologies allow for micrometric precision in intraoperative gap balancing [11]. Most commercially available robotic systems provide a touchscreen with integrated software, controlled directly by the surgeon, capable of extreme accuracy (μm) during intraoperative gap determination.

The current results also challenged the recent concept that the reproduction of the constitutional knee alignment in its extreme variants represents the main driver to a close-to-normal knee kinematics and joint proprioception: if the reproduction of the joint obliquity through pre-planned bone cuts has a major role on the coronal and transverse plane alignment of the knee joint at initial contact [12], the restoration of pre-arthritic, “static joint” line is not sufficient to recover the real, pre-arthritic kinematics of knee joint, which is “dynamic” by definition. Multiple studies [13–16] confirmed that the static measures in frontal radiographs are not necessarily a casual predictor of the dynamic contact loads in vivo. The uKA with its “pure resurfacing” principles has multiple fascinating features: it is applicable using standard instrumentation upgraded with 2-mm shims to compensate for the predicted cartilage wear, does not require advanced forms of radiologic evaluation (i.e., CT scans) and it is independent from the use of enabling technologies. This “one size fits all”, pure measured resection surgical technique, anyway, is based on the reproduction of a rigorously symmetric extension gap, to a point that the original authors recommend a 2-mm bone recut (especially in originally varus knees) to ensure the symmetry of the gap itself [9] if a slight asymmetry is intraoperatively noted. Edelstein et al. [17] recently published a worrisome report on the relationship between laxity, balance, and alignment following uKA TKA: in 382 simulated TKAs, only less than 30% had a mediolateral extension ligament balance within ± 1 mm and up to 56% had a medial flexion gap looser than the lateral one.

The current study also showed that major differences still exist between the normal knee (as shown in the control group) and both TKA groups. Previous studies, including those from the senior author’s institution [18, 19], showed strong kinematic differences when knees were evaluated during the stance phase of gait (center of rotation being on the lateral knee compartment) or during the swing phase of gait, like stairs ascending activities and squatting, having the center of rotation on the medial compartment. This normal kinematics was guided by the anterior cruciate ligament (ACL), which is routinely removed during TKA. Interestingly, what has been proposed as the gold standard for many years, a cruciate sparing TKA design combined with an MA surgical technique, showed inferior kinematics when compared with the same design in combination with a KA surgical technique [1], highlighting the role of the alignment in TKA kinematics.

The current study also showed that a final HKA alignment ± 3° might play a minor role in postoperative knee kinematics. In fact, no statistically significant differences in final HKA alignment were found between uKA and simplified KA cohorts, suggesting that the statistical differences between patients in those cohorts were mainly due to the different balancing techniques. On this topic, Vendittoli et al. [20] showed that 51% of knees could be treated indiscriminately with a uKA [9] or restricted-KA surgical technique and had a final HKA ± 5°.

Another interesting finding of the current study was the ROM determined in the contro-lateral, healthy knee. In both cohorts (uKA and simplified KA), the healthy knee ROM during the stance and swing phases of gait was similar to the surgical knee. This finding confirmed previous reports [21] showing a “symmetrical gait” between the surgical and non-surgical knee in patients who underwent TKA. The implementation of these compensatory strategies in the non-operated side may subsequently result in altered joint loading and progression of OA in the non-operated knee.

The current study has several limitations. First, this is a single institution study analyzing three small cohorts of patients and the conclusions of this study may not apply to larger cohorts. Second, two different medially-stabilized designs were used in this study. This was done because of the lack of availability of a robotic system for the uKA knee system used in the study (GMK Sphere, Medacta, Castel San Pietro, Switzerland). Different designs with different geometries may result in different findings. Third, a clear definition of an optimal intercompartmental gap difference in flexion as well as in extension has not been established yet and the authors proposed their own. Fourth, the surgery was performed under regional anesthesia using a tourniquet, and the resulting kinematics could be altered. However, it has been reported that this measurement system had satisfactory reproducibility [22]. Finally, detailed clinical outcomes have not been reported but all TKA patients had a score > 80 on the Knee Society Clinical Score scale.

## Conclusion

In summary, patients with a slight increase in the lateral laxity, in extension as well as in flexion, showed closer to normal knee kinematics. This study, one more time, showed that modern surgical techniques, improved enabling technologies, and new-generation implant designs cannot inherently reproduce normal knee kinematics following TKA. However, the current study was the first to define slightly asymmetric targets during intraoperative knee balancing with the help of advanced technologies: the clinical benefit of this robotic-assisted, surgical approach needs to be proved by further studies.

## Data Availability

The datasets used and/or analyzed during the current study are available from the corresponding author on reasonable request.
